# Microalgae-based bioremediation of refractory pollutants: an approach towards environmental sustainability

**DOI:** 10.1186/s12934-024-02638-0

**Published:** 2025-01-14

**Authors:** Mostafa M. El-Sheekh, Hala Y. El-Kassas, Sameh S. Ali

**Affiliations:** 1 Botany Department, Faculty of Science, Tanta University, Tanta, 31527 Egypt; 2 National Institute of Oceanography and Fisheries, NIOF, Alexandria, 21556 Egypt

**Keywords:** Microalgae, Bioremediation, Environmental pollutants, Sustainability, Nanomaterials, Biorefinery

## Abstract

Extensive anthropogenic activity has led to the accumulation of organic and inorganic contaminants in diverse ecosystems, which presents significant challenges for the environment and its inhabitants. Utilizing microalgae as a bioremediation tool can present a potential solution to these challenges. Microalgae have gained significant attention as a promising biotechnological solution for detoxifying environmental pollutants. This is due to their advantages, such as rapid growth rate, cost-effectiveness, high oil-rich biomass production, and ease of implementation. Moreover, microalgae-based remediation is more environmentally sustainable for not generating additional waste sludge, capturing atmospheric CO_2_, and being efficient for nutrient recycling and sustainable algal biomass production for biofuels and high-value-added products generation. Hence, microalgae can achieve sustainability's three main pillars (environmental, economic, and social). Microalgal biomass can mediate contaminated wastewater effectively through accumulation, adsorption, and metabolism. These mechanisms enable the microalgae to reduce the concentration of heavy metals and organic contaminants to levels that are considered non-toxic. However, several factors, such as microalgal strain, cultivation technique, and the type of pollutants, limit the understanding of the microalgal removal mechanism and efficiency. Furthermore, adopting novel technological advancements (e.g., nanotechnology) may serve as a viable approach to address the challenge of refractory pollutants and bioremediation process sustainability. Therefore, this review discusses the mechanism and the ability of different microalgal species to mitigate persistent refractory pollutants, such as industrial effluents, dyes, pesticides, and pharmaceuticals. Also, this review paper provided insight into the production of nanomaterials, nanoparticles, and nanoparticle-based biosensors from microalgae and the immobilization of microalgae on nanomaterials to enhance bioremediation process efficiency. This review may open a new avenue for future advancing research regarding a sustainable biodegradation process of refractory pollutants.

## Introduction

Environmental contamination due to human activity has worsened recently, posing a severe ecological and public health threat [1–3]. Water bodies have been severely contaminated by various organic pollutants (e.g., dyes, phenols, pesticides, medicines, and hormones) released during human activities. Many of these pollutants can build up in organisms and negatively affect metabolism, growth, and development [4, 5]. Furthermore, most refractory organic pollutants have been proven to induce diseases like cancer, cardiovascular conditions, and reproductive issues [6–8]. Discharging wastewater contaminated with toxic and hazardous organic compounds from industrial plants poses significant environmental challenges [9, 10]. Wastewaters generated during various industrial processes frequently contain toxic organic compounds that are not easily treatable through direct biological means. Treating this industrial wastewater is necessary to comply with the prescribed standards for its discharge or reuse within the industrial process [11–14].

**Fig. 1:**
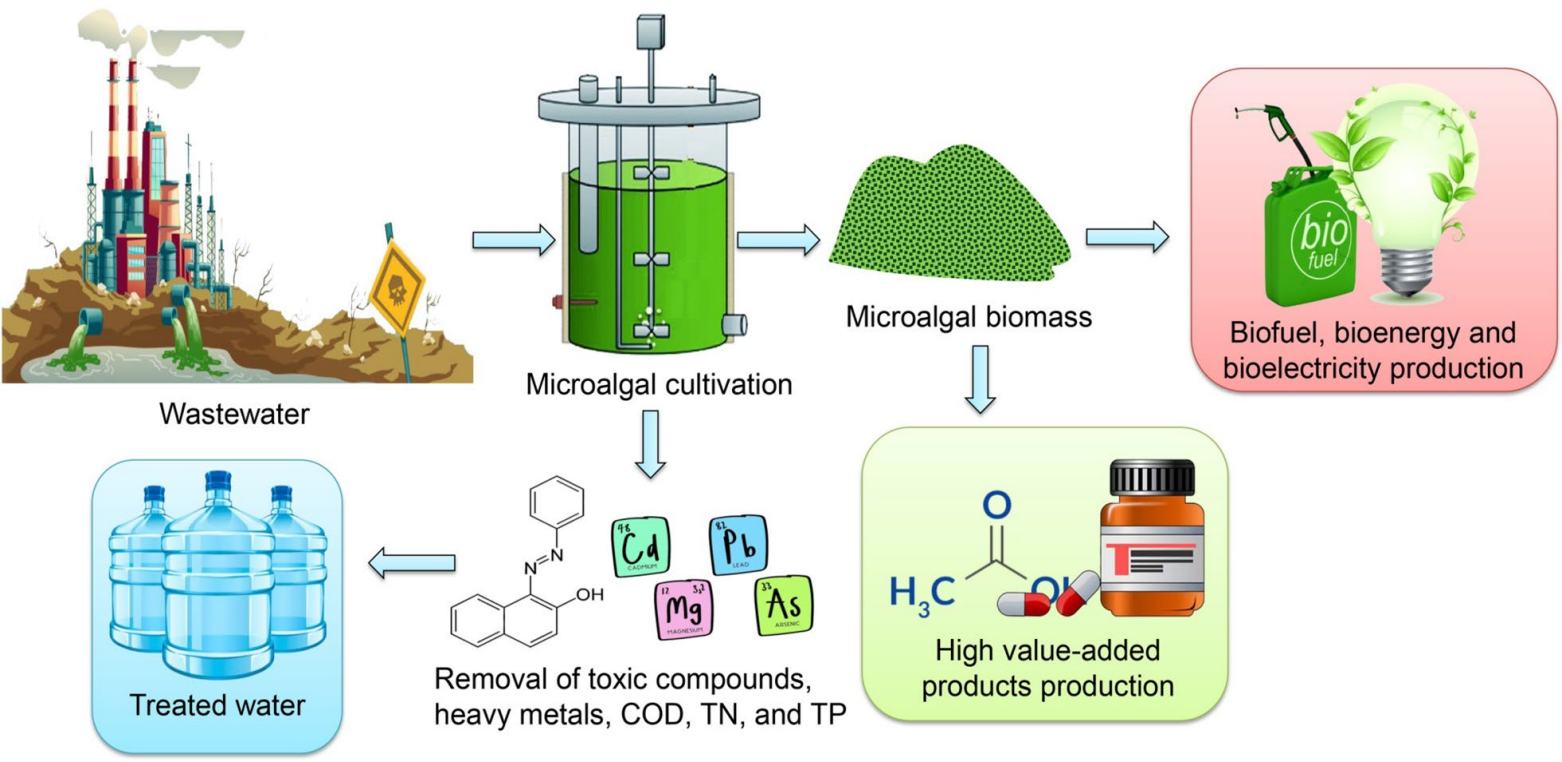
Schematic diagram of wastewater treatment for contaminants removal and high value-added product generation by microalgae

Industrial effluents exhibit a high degree of persistence and possess biotoxic properties [15]. Hence, it is imperative to employ measures to mitigate these organic pollutants' presence before their discharge into an aquatic ecosystem. Physical–chemical wastewater treatment costs are typically high within the industrial sector, particularly in developing countries [16]. Furthermore, it should be noted that secondary effluents within wastewater treatment systems are known to contain various components (e.g., inorganic nitrogen, phosphorus, persistent organics, and released heavy metals). These constituents have been found to contribute to long-term challenges and concerns. In recent years, the significance of low-cost biological wastewater treatment with algae has been emphasized as a viable alternative to conventional wastewater treatment methods [17]. Bioremediation is a highly effective approach for the removal of pollutants in terrestrial and aquatic environments (Fig. 1). Several examples of the necessary bioremediation agents, including *Acalypha indica*, *Saccharomyces*, *Aspergillus*, and algae [18–20]. Green technology has gained significant popularity in recent years owing to its substantial environmental benefits. These advantages include cost-effectiveness, absence of secondary pollution, superior efficiency, and the utilization of eco-friendly materials [21].

Microalgae cultures play a vital role in the wastewater treatment process because they produce biomass suitable for diverse applications, their potential as a solution for tertiary biotreatment, and their capacity to eliminate organic contaminants from wastewater. According to Xiong et al. [22], their study demonstrated the efficient biodegradation of carbamazepine by *Scenedesmus* sp. Furthermore, they observed a significant inhibition of microalgal growth, even at a high concentration of carbamazepine (100 mg/L), resulting in a 30% reduction. In addition, microalgae exhibit remarkable resilience in the face of elevated levels of refractory organic pollutants, including antibiotic industry waste, cyanide, dyes, and pesticides. These pollutants are known for their persistence and high toxicity [23].

Bioprocess should be sustainable and contribute to the circular bioeconomy and a cleaner society achieving the sustainability requirement (social, economic, and environmental pillars) [24]. The main obstacles to algae growth promotion are opaqueness, high polymeric substances (long peptides, lactose), and suspended solids. Microalgae-based remediation is more environmentally sustainable for not generating additional waste sludge and being able to capture gaseous CO_2_ [25]. Moreover, they are efficient for nutrient recycling and sustainable algal biomass production for biofuels [26]. The algal process can curb their greenhouse gas emissions effectively rather than any other microorganism-based bioprocess, improve carbon footprints, and make the process environmentally sustainable. Fast-growing microalgae-based bioprocess is more fascinating due to its carbon reduction and sustainability features [27]. The implementation of microalgal bioremediation approaches may bring benefits such as (i) Improvement of carbon footprint and efficient wastewater treatment, (ii) Generation of microalgal biomass and lipid as feedstock/precursor for various high-value-added product and biofuel productions, and (iii) Development of a sustainable algae process for environmental remediation as well as for circular bioeconomy [27, 28]. Therefore, exploring advanced techniques by utilizing microalgae for refractory pollutants mitigation from wastewater with microalgae cultivation to achieve a sustainable bioprocess to avoid any successive pollution from reprocessing of produced algal biomass for further biotechnological applications is essential to be discussed. This coupling feature of microalgae holds great promise for the sustainable development of any industrial bioprocess and environmental bioremediation.

Numerous studies have recently been conducted on immobilization techniques and immobilized biomass materials to remediate refractory organic wastewater [29, 30]. Bag et al. [31] disclosed that there are a variety of immobilization techniques used to immobilize microalgae, such as capturing cyanobacteria in the matrix (e.g., agarose, carrageenan, chitosan, alginate, and polyurethane foam). Using immobilized cells has been investigated to treat effluents containing phenols, rubber press wastes, distillery waters, olive oil mill wastes, paper mill sediment, dairy wastewaters, and textile dye effluents [32]. However, it has been discovered that these techniques have little activity to decompose organic and inorganic substances found in large quantities in discharges. Numerous studies have demonstrated the exceptional properties of nanomaterials (NMs), including enhanced catalysis and adsorption properties, as well as their high density [33–38].

Environmental factors (e.g., photoperiod, light intensity, pH value, temperature, carbon dioxide, and salinity) play a vital role in biomass production in huge quantities [39]. These environmental factors affect the microalgal metabolic activity of microalgae biomass production and their bioremediation efficiency [40]. Several studies investigated various techniques to enhance microalgal growth by designing a favorable range of environmental factors to improve their growth rate. However, these factors vary according to microalgae species and strain [41]. Light is the primary energy source used by microalgae for the photosynthesis process. Hence, the lighting level (natural or artificial), including the light intensity and period, enhances the microalgal growth until it reaches the maximum rate. For instance, the maximum light intensity for *Scenedesmus obliquus* 276.7 [42], *Dunaliella* sp. [43], and *Botryococcus braunii* UTEX 2441 [44] was 150, 61, and 400 μmol/m^2^/s, respectively. Most microalgae have various light exposure period requirements because of their natural habitat, microalgae species, and growth conditions. Salinity refers to the salt content in the water for microalgal cell growth. Expectedly, marine algae use or consume higher salinity concentrations than freshwater algae [45]. Salinity is also a critical parameter to be explored, as salinity may affect algae growth and algae cell biochemical composition [45]. Freshwater algal cultivation in high salinity may negatively impact the microbial cell structure [46]. High salinity may inhibit the photosynthesis process and decrease the biomass production. For instance, the optimum salt concentration for the growth of *Thalassiosira weissflogii*, Artic-sea-ice algae, *Isochrysis* sp., *Nannochloropsis oculata*, *Nitzschia* was 25, 4–74, 20–30, 10–15 psu, respectively [47, 48]. Moreover, temperature is another essential environmental factor controlling microalgal growth. Similar to the effect of photoperiod and light intensity, microalgae growth increases linearly to an optimal point, after which cell growth gradually declines [49]. Moreover, temperature affects microbial cell size and metabolic activities [50]. Normally, the ideal temperature range for microalgae growth at optimal conditions is between 20 °C and 35 °C [51].

Microalgal-based systems are acknowledged as an alternative way for the treatment of wastewater. In addition to the removal of nutrients, these systems allow for the development of microalgal biomass, which may then be utilized as a raw material for the manufacture of bioenergy and biochemicals. However, prior research has infrequently illustrated the actual applications of algal-bioremediation systems for emerging contaminants in laboratory settings [52–54]. Conversely, no effort has been made to provide a thorough elucidation of the mechanisms behind algal bioremediation systems, as well as their benefits and drawbacks. Certain investigations have indicated that various contaminants that exhibit increased algal-bioremediation in laboratory settings only achieve partial removal during pilot-scale evaluations [55]. Microalgal bioreactors that are used on a commercial scale often operate with shorter hydraulic retention durations and make use of processes that are either continuous or semi-continuous [56]. Hence, there is a significant obstacle that must be overcome in order to validate the removal capabilities of pollutants that were discovered through research conducted on a laboratory scale and then applied on a pilot scale. A limited number of studies have assessed the efficacy of pollutant removal from real wastewater. The disparity between experimental findings and real applications renders the commercial utilization of algae bioremediation for contaminants remain unclear. A systematic approach was implemented to identify pertinent literature from databases such as PubMed, Scopus, Web of Science, and Google Scholar in order to guarantee a comprehensive review. Articles were selected based on a variety of criteria, such as the topic’s relevance, the publication date, and the presence of substantial reviews or experimental data on algal-based bioremediation of refractory pollutants. The methodology for this review paper was restricted to articles written in English and encompassed data identification and extraction, data screening, and eligibility analysis, covering the period from 2014 to 2024. The search was performed utilizing the following keywords: “ algal bioremediation”, “emerging pollutants”, “wastewater treatment”, “algal bioreactors”, “recalcitrant pollutants”, “pharmaceuticals”, “microalgal-based nannotechnology”, “synthetic dyes”, “refractory compounds”, and “industrial effluents”.

The present review paper provides a comprehensive examination of the pivotal function of microalgae, which are highly prevalent in natural environments and possess the capacity to serve as a reservoir for contaminants. Additionally, the paper explores the potential of diverse species of algae to mitigate the burden of pollutants originating from different sources of wastewater effluents. Furthermore, it is important to emphasize the application of nanotechnology in bioremediation to address environmental pollutants.

## Algal bioreactors for bioremediation of refractory compounds

Refractory materials are extensively dispersed in nature and often employed in industrial operations. The term “refractory compounds” is a relative term since it relies on the process of degradation that is being employed [57]. It is frequently impractical because microorganism degradation requires extremely extensive treatment durations. According to used treatment techniques, a chemical is regarded as refractory if the breakdown rate under aerobic circumstances is lower than 80% [58].

Aromatic and polyaromatic compounds comprise many refractory materials [59]. The derivatives of several polyaromatic hydrocarbons are also refractory. At least four condensed aromatic rings make up each one. Due to their extreme hydrophobicity, they rarely dissolve in water. Since they have a large molecular weight, they cannot pass through cell membranes [60]. They must first be partly destroyed extracellularly, either by enzymes or chemically, by oxidation, before mineralization. Lignin is the source of yet another class of refractory substances [60]. Only monoaromatic ring structures joined together primarily by ether-bridges or aliphatic linkages make up higher molecular weight lignin derivatives. These lignin fragments have molecular weights and are found in effluent from the pulp and paper industries [61]. Compounds produced from lignin called humic acids are often found in agricultural wastewaters.

Certain microalgal species can degrade these substances, as shown in Table 1 [62–81]. With a molecular weight of 20 kDa, polyethylene glycols (PEGs) are a significant class of synthetic, non-ionic, water-soluble chemicals. Degradation becomes more challenging with increasing molecular weight. The biological degradation of higher molecular weight PEGs is difficult [82]. Additionally, many industrially relevant low molecular weight xenobiotic chemicals are also resistant and toxic. Aromatic sulfonates, mostly used as surfactants and dyes, are another intriguing class of xenobiotic chemicals with a somewhat refractory property [83]. As a result of their direct environmental importance, efforts focus primarily on replacing refractory substances with readily biodegradable ones. Slow reaction rates at extremely low concentrations are another issue for biological degradation. Despite often observed strong affinity degradation, first-order traits become dominant at low enough concentrations. However, biofilm systems greatly boosted refractory compound degradation rates [84].

**Table 1:** Removal efficiency of refractory pollutants by different microalgal species

Algal species	Refractory pollutants	Removal efficiency (%)	References
*Chlorella vulgari*	Hg	72.9	[62]
*Chlorella vulgaris* and* Spirulina platensis*	Cr(III)	85	[63]
*Chlamydomonas mexicana*	Textile azo dyes (Red HE8B, Reactive Green 27, and Acid Blue 29)	39%–64%	[64]
*Pterocladiella capillacea*	Chromium	100	[65]
*Nannochloris* sp.	Sulfamethoxazole	99	[66]
*Scenedesmus* sp.	Chromium	60	[67]
*Chlorella sorokiniana*	Cr(III)	96	[68]
*Scenedesmus* sp.	Cr(VI)	100	[69]
*Chlamydomonas* sp.	Sulfadiazine	54	[66]
*Chlorella vulgaris*	Levofloxacin	91	[66]
Swedish microalgae	Caffeine	< 40	[66]
*Scenedesmus quadricauda*-based biochar	Cr(VI)	93	[70]
*Selenastrum capricornutum* and *Scenedesmus acutus*	Benzo(a)anthracene and benzo(a)pyrene	85–90	[71]
*Chlorella* sp. MM3	Pyrene	100	[72]
*Chlorella* sp.	Amoxicillin	99.3	[73]
*Chlorella regularis*	Amoxicillin	88	[74]
*Chlorella pyrenoidosa*	Cefradine	75.4	[75]
*Spirulina platensis*	Chlortetracycline	99.5	[76]
*Chlorella pyrenoidosa*	Roxithromycin	45.9–53.3	[77]
*Chlorella* sp.	Chlorpyrifos	100	[78]
*Scenedesmus* sp.	Chlorpyrifos	75	[78]
*Hapalosyphon* sp.	Chlorpyrifos	50	[78]
*Scenedesmus *sp. TXH	Imidacloprid	71.2	[79]
*Scenedesmus quadricauda*	Bromacil	94	[80]
*Scenedesmus quadricauda*	Atrazine	83	[80]
*Scenedesmus quadricauda*	Diuron	88	[80]
*Chlorella* sp.	Imidacloprid	57–62%	[81]

Humic acids are one example of a naturally occurring or naturally generated refractory substance that is frequently nontoxic and typically does not harm the environment [85]. Humic acids stay in natural settings for a long time but do not accumulate and finally become mineralized. Humic acids could still be an issue whether in wastewater or the water for reuse. Humid acid complexes comprise roughly 25% of the total organic carbon in the soil and about 50% of the total organic carbon in water [86]. The pulp and paper industries’ effluent contains substances similar to lignin. They cannot be recycled without treatment, nor can they be discharged into recipient waters. It is uncommon to see 100% degradation in wastewater treatment, especially municipal wastewater. These residual organic contaminants are probably at least somewhat refractory. Therefore, enhancing the growth condition and the cultivation techniques may improve the biodegradation rate of refractory pollutants.

**Fig. 2:**
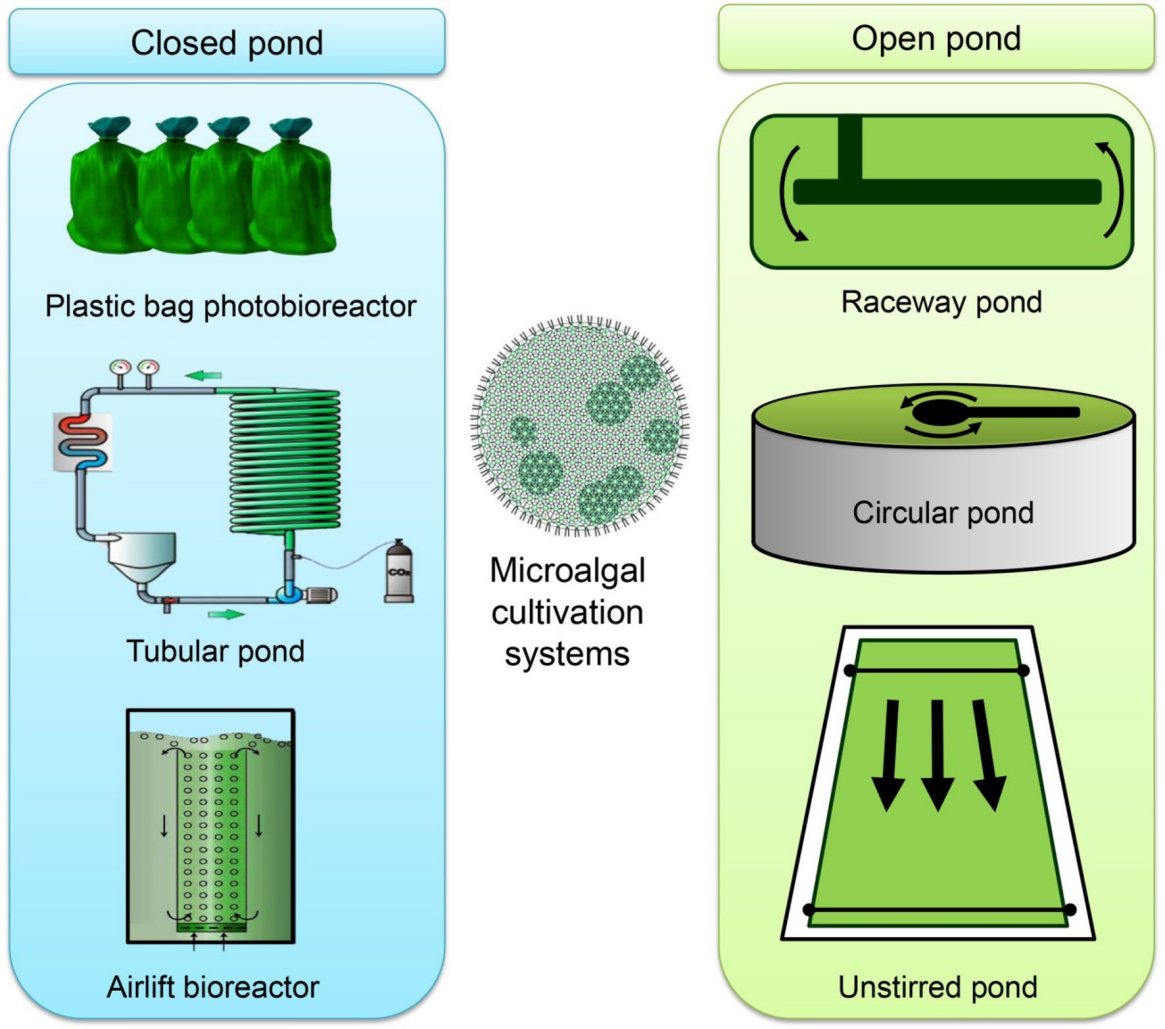
Different types of microalgal cultivation systems

Photobioreactors (PBRs) applied in the bioremediation of pollutants are generally divided into closed and opened systems, as shown in Fig. 2, each with advantages and disadvantages of their own. Open systems include raceway tanks, scrubbers, open ponds, and ponds, whereas closed systems consist of flat plate PBR and tubular PBR (bubble and airlift mechanism) [87]. Additional technological optimization in the PBRs is necessary to make microalgae useful for wastewater treatment. Innovative methods that may be applied to monitor, automatically control, and accurately anticipate microalgae output are urgently needed. This has led to a significant amount of research being done on novel Internet of Things (IoT) applications in microalgae biorefinery [88]. IoT could be used in a microalgae biorefinery to automate microalgae cultivation, monitor and adjust microalgal cultivation parameters, increase microalgae productivity, identify toxic algae species, screen for target microalgae species, categorize microalgae species, and detect the viability of microalgal cells [89, 90]. Recently, Roostaei et al. [91] concluded that to better monitor the environment and address issues with data latency, energy use, and bandwidth cost, this pilot research investigated several applications of the IoT computing architecture. This work indicates that different types of sensors and boards may be utilized to build edge computing-based IoT-based sensor networks, which lower the energy and bandwidth for data transmission and reaction times in addition to cost reduction. Moreover, Oruganti et al. [92] stated that Algorithms based on artificial intelligence (AI) and machine learning (ML) provide creative methods for identifying, forecasting, and controlling risks in the biorefinery and treatment of aqueous emulsions from algae. Therefore, AI/ML usage in algal cultivation assists in effective decision-making. Application of ML tools in algal biorefinery helps increase product yield and novel deep-learning ML algorithms incorporating large databases are needed.

The algae are crucial for biomonitoring and regulating organic contaminants in aquatic habitats. There has been substantial research on the biological extraction and bioremediation of heavy metals and organic pollutants using higher plants and microbes [93]. The role of microalgae to mitigate aquatic environments contaminated by organic matter cannot be ignored. For instance, Matamoros et al. [94] found that a variety of emerging organic contaminants (EOCs) could be removed from urban wastewater using microalgae-based wastewater treatment systems, such as  high rate algal ponds (HRAPs). The authors found that HRAPs can remove > 90% of acetaminophen, caffeine, hydrocinnamic acid, ibuprofen, and methyl dihydrojasmonate. HRAPs can also remove 60–90% of galaxolide, naproxen, octylphenol, oxybenzone, tonalide, tributyl phosphate, and triclosan, bisphenol A. In addition, HRAPs can remove 40–60% of atrazine, benzotriazole, cashmeran, diazinon, celestolide, diclofenac, OH-benzothiazole, and triphenyl phosphate. Moreover, 30% of 2,4-D, benzothiazole, carbamazepine, methylparaben, and tris(2- chloroethyl) phosphate can be removed by HRAPs. However, low temperature can negatively impact the elimination of emerging pollutants in HRAPs. The ecotoxicological risk assessment study found that the hazard quotient indexes for the influent wastewater were eliminated by up to 90%, indicating no acute toxicity risk linked with the examined EOCs at the water effluents. The wastewater treatment systems that use microalgae may be split into two categories: (i) immobilized microalgae systems and (ii) suspended microalgae systems [95].

### Open systems

Open ponds are the most widely used system for microalgal production, as they were the first to be developed and are still in use today. Open pond systems consume less energy and likely have lower construction and maintenance costs [96]. Pond systems are often used in reactor systems for the culture due to their low price and straightforward construction of microalgae and wastewater treatment [97]. Pond systems are constrained by the lack of light, temperature fluctuations, poor mixing, and limited productivity of microalgal biomass. Due to the C/N/P imbalance, the CO_2_ supply is inconsistent and heterogeneous, reducing microalgal biomass productivity [98]. However, better mixing, HRAPs, paddle wheel stirrers, and enough gas intrusion can help overcome some limitations. Using improvised aeration and CO_2_ delivery can increase biomass productivity and the rates at which different contaminants are eliminated [99].

Due to the limited control over pollution and growth conditions, only microalgae that can withstand harsh environmental conditions can be cultivated in these bioreactors [100]. Open raceway PBR systems are the most prevalent microalgal culture systems on a commercial scale; they consist of a circuit of parallel channels where paddle wheels promote microalgal circulation. Raceway PBR typically functions at depths between 15 and 30 cm and flow rates between 15 and 30 cm/second [96, 101]. Open raceway PBRs utilized less energy than other open ponds and confined PBRs. The raceway PBR is one of the most researched microalgal culture systems. This bioreactor configuration has been subjected to several experiments, particularly in terms of improved mass transfer, enhanced mixing efficiency, and light availability [102]. Thin-layer cascades are another bioreactor design utilized for microalgal culture. After flowing from the top to the bottom along a sloping surface and accumulating in a retention tank, the culture is pushed to the top in these systems, causing considerable turbulence. The depth of culture in thin-layer cascades ranges from a few millimeters to two centimeters, while the slope ranges from 1 to 3% [103]. These reactors utilize light more efficiently than conventional configurations, resulting in a higher biomass concentration and increased production [104]. Masojídek et al. [105] evaluated the productivity of *Chlorella* spp. in thin-layer units with varying depths (6 mm) and capacities (170 and 2200 L). The 170 L unit had a larger ratio of exposed surface to total cultivation volume (S/V) (133/m), which resulted in greater biomass productivity (19 g/m^2^/day). A model of the culture's hydrodynamics revealed a highly turbulent flow that allowed for numerous cycles of light and darkness. To overcome the wastewater turbidity on the photosynthesis of microalgae.

Multilayer bioreactors are an open PBR configuration consisting of numerous open containers of varying heights [106]. In these systems, gravity circulates the culture between the upper and lower vessels before pumping it back to the upper tank. This layered method has the advantage of requiring less space for installation [96]. Min et al. [107] evaluated a multilayer system with a 7500 L capacity for generating *Chlorella* sp. biomass in conjunction with wastewater treatment. The system consisted of four 1800 L containers, a mixing tank, a pump, a CO_2_ tank, and a pH controller. The photobioreactor was housed in a greenhouse tunnel to reduce environmental variations.

**Fig. 3:**
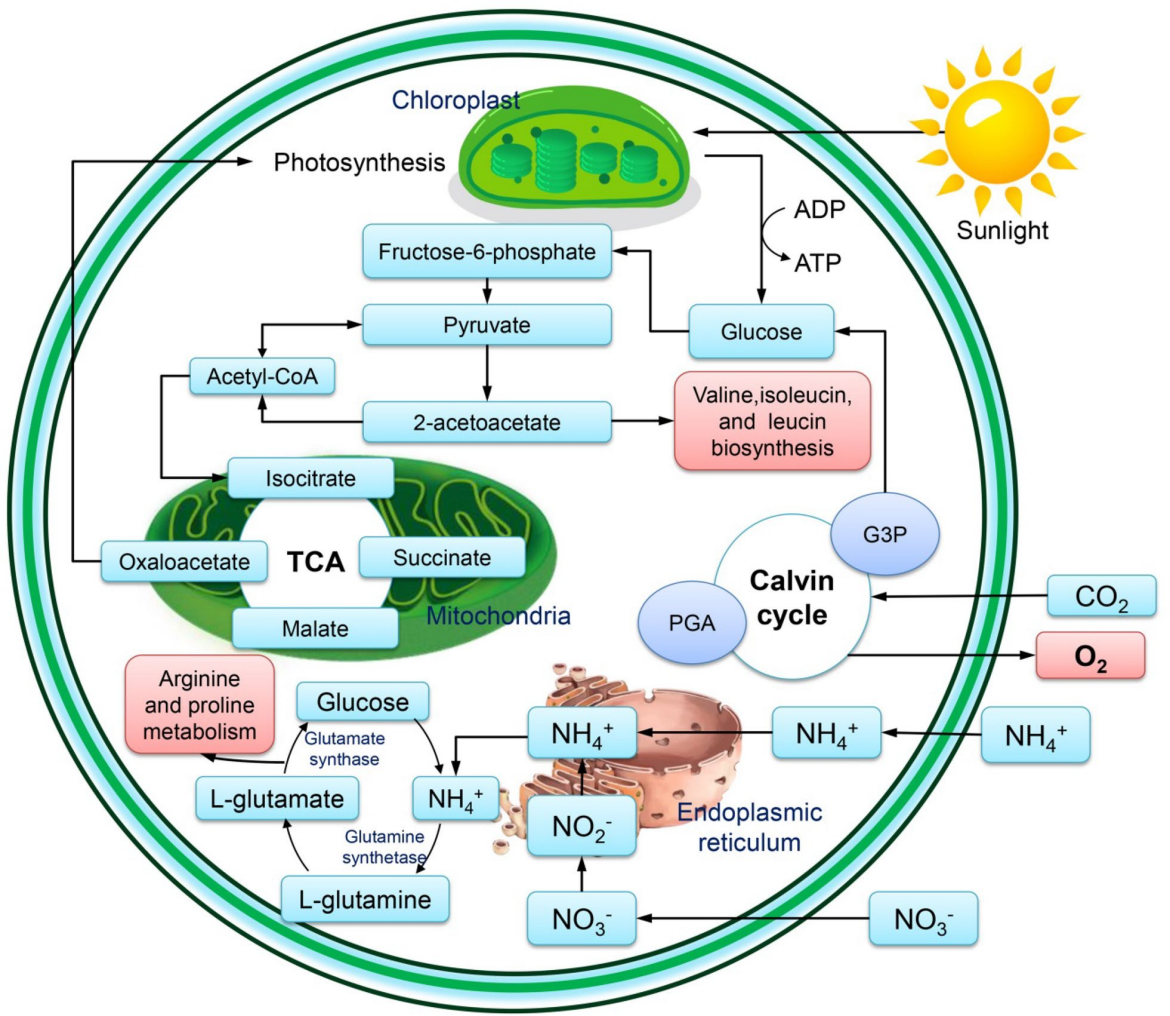
Nitrogen removal during the wastewater treatment by microalgae

**Fig. 4:**
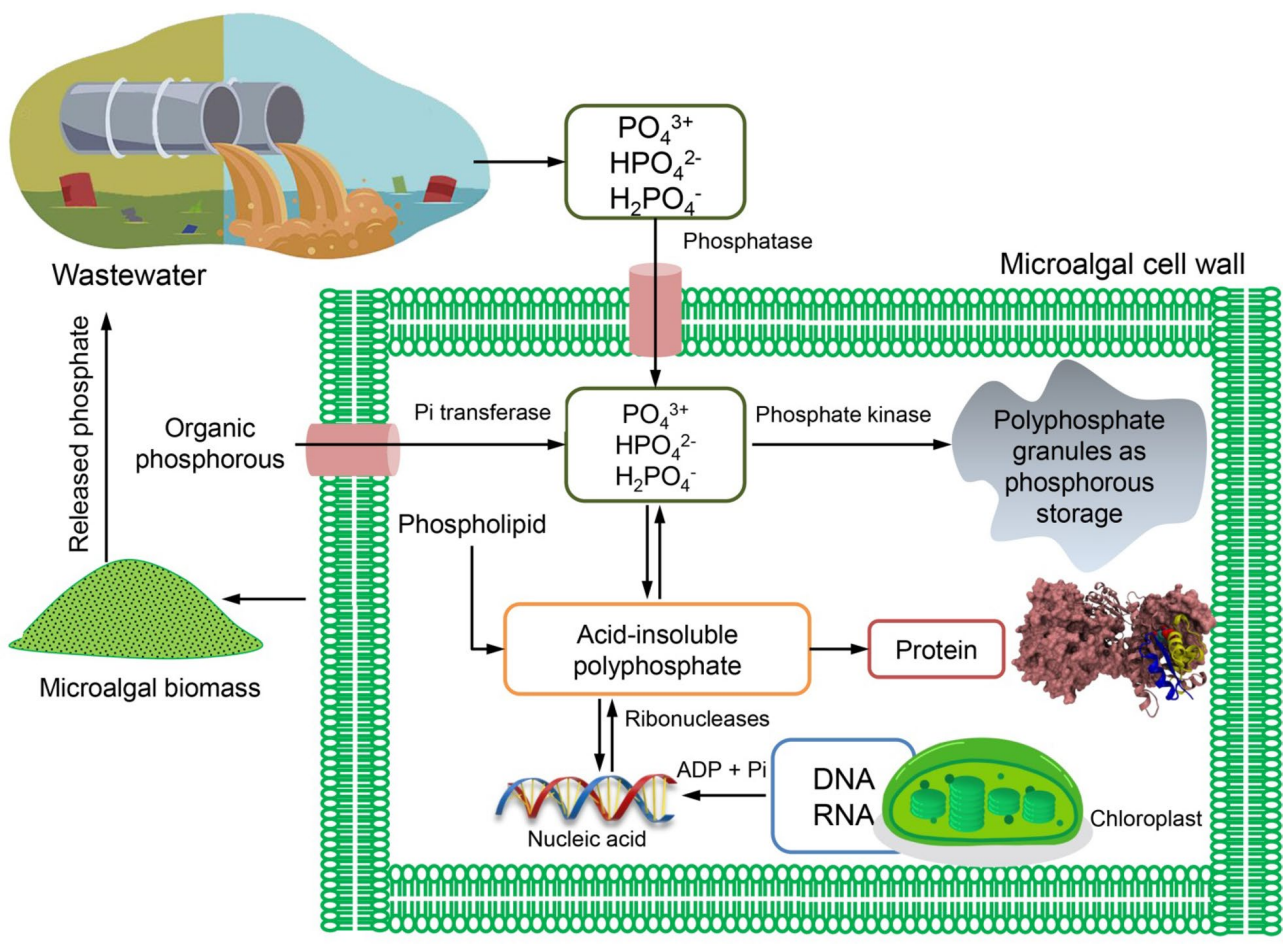
Phosphorous removal during wastewater treatment by microalgae

The removal of N and P by microalgae-suspended systems typically ranges from 10 to 97% based on the parameters of the procedure, the type of microalgae used, the cultivation method, the characteristics of the effluent, and the tank size [108]. Due to microalgae’s ability to remove nutrients and contaminants, microalgae-based wastewater treatment (MBWT) is one of the most desirable methods. Removal of COD, HMs, and ECs from wastewater, as well as nutrient removal and recoveries (e.g., total nitrogen) (Fig. 3) and total phosphorus (Fig. 4), are all examples of bioremediation, water recovery, and reusability of culture medium are a few advantages of MBWT [109, 110].

### Closed systems

Compared to open pond systems, closed systems offer superior opportunities for efficient light distribution and mixing (bubble or airlift systems), boosting biomass productivity and removal efficiency [106]. PBRs are sophisticated closed biorefinery plants designed to use wastewater to create reusable water and various value-added products, biofuel, and bioenergy. PBRs should be easy to use, affordable, productive in volume, energy-efficient, and adaptable to industrial applications [111]. A simplified PBR model should have high light-harvesting capacity to ensure that microalgal species can be transported, channeled, and distributed for biomass production; permit practical maintenance of operational parameters to encourage the cells for high light energy utilization; and require less operating expense. Xiaogang et al. [112] reported that biomass productivity in closed PBRs ranges from 2 to 3 g/L/day, or 0.73 to 1.05 tons (dry biomass) m^3^/year. Min et al. [113] found that COD in closed PBRs is reduced by 70% when *Chlorella* spp. is grown on urban wastewater. PBRs achieve excellent biomass productivity while removing the risk of evaporation and contamination. Rosli et al. [114] found that *Synechocystis* sp. in shrimp wastewater produced 500 mg/L of biomass using organic molecules. *Synechocystis* sp. recovered 20.2 mg/g dw/day phosphate on the 1st day and also recovered nitrite (67.9%), nitrate (80.1%), phosphate (96.99%), and ammonium (98.1%).

On the other hand, PBRs need higher capital expenditures, challenging scaling-up, and significant shear stresses [115]. Investing in creating economically practical PBR designs with better operations is worthwhile due to the high biomass output and regulated limiting variables in PBRs. Full-proof PBR design is still required for large-scale microalgal culture despite extensive study. The primary element affecting PBR arrangement in microalgal production is the effect of surface-to-volume ratio on light penetration. The most admired PBR design, including stirred tank, airlift, and bubble column PBRs, is the tubular PBR. In cultivation conditions, tubular design significantly reduces the possibility of contamination [116]. These reactors have a particular arrangement of transparent tubes built-in spiral, straight, and curved designs.

Stirred tanks used in manufacturing medicines or fine chemicals are the most typical form of PBRs [117]. These PBRs guarantee that operational parameters are regulated and that sterile conditions are maintained for microalgal culture. Due to their enormous surface area and minimal contamination risk, vertical tubular PBRs are the most practical, portable, and valuable reactors for outdoor microalgal production [118]. In these systems, transparent vertical tubes are implanted for significant light penetration. Gouveia et al. [119] stated that cultivating *Scenedesmus obliquus* and *Chlorella vulgaris* in pilot-scale vertical tubular PBR (150 L) can remove 64% of COD. Moreover, bubble column reactors are frequently used to manufacture culinary items, including vinegar, beer, and baker’s yeast, as well as commercial-scale wastewater treatment. A simple bubble column design calls for a height two times greater than the diameter [112]. Except for the sparger, no other internal structure is incorporated into the design. Pricked horizontal plates are mounted to disseminate the mixed bubbles produced from the sparger. PBRs’ mass transportation and column hydrodynamic properties are determined by the sorts of bubbles that arise from the sparger [112].

## Systems using immobilized microalgae to treat wastewater

The utilization of immobilized microalgae in biotechnology holds significant potential for addressing refractory pollutants mitigation challenges. The concept of “immobilized microalgae” originated from the well-established concept of “immobilization,” as described by Tong and Derek [120]. Microalgal immobilization refers to a biotechnological process that employs both natural and artificial physical and chemical methods to hinder the autonomous movement of living microalgal cells within their native environment [121]. Confining microalgal cells to a restricted space can preserve their desired biological functionality, allowing for subsequent reuse within an aqueous phase system. Due to its high population density, the integration of high-efficiency treatment and subsequent separation in microalgal immobilization can reduce the carbon footprint and enhance treatment efficiency without the need for additional energy-intensive recycling procedures [122]. In addition, it is worth noting that immobilized microalgal cultivation exhibits comparable potential for nutrient recovery when compared to suspended microalgal cultivation. Moreover, it has a greater commercial value in the chemical and agricultural sectors. The utilization of immobilized culture has gained recognition as a viable approach for the remediation of aquatic environments and the enhancement of sustainable biological wastewater treatment [123].

Immobilized microalgae techniques are widely recognized and extensively employed for macronutrient removal and biomass augmentation [124]. In recent years, there has been a notable shift in the approach to wastewater treatment. The focus has transitioned from solely removing pollutants to adopting a collaborative approach involving both pollutant removal and recovering valuable resources from sewage [125, 126]. This shift has led to a significant rise in research interest in treating microalgal and immobilized microalgal wastewater. Nevertheless, the progress in microalgal immobilization seems to be comparatively sluggish. There is a need to establish a connection between the microalgal immobilization process and wastewater treatment. There is a scarcity of information regarding immobilization technology and the efforts to eliminate contaminants. The current methods employed for microalgal immobilization technologies and removing pollutants by immobilized microalgae exhibit certain deficiencies, potentially constraining the widespread implementation of immobilized microalgal systems for enhancing the microalgal wastewater treatment process [120]. According to an extensive review of the literature, the utilization of immobilized microalgae has been recognized as a promising approach for the treatment of wastewater using microalgae [17, 124]. The concurrent enhancement of wastewater treatment and microalgal biomass recovery can be achieved by developing immobilized microalgal technology. Furthermore, exploring methodologies aimed at facilitating the large-scale production of immobilized microalgal systems holds considerable potential in expediting the utilization of such systems in engineering applications.

Microalgae possess remarkable attributes, including self-aggregating and producing extracellular materials [127]. These properties enable microalgae to adhere to both living organisms (biotic surfaces) and non-living substances (abiotic surfaces) within porous structures, thereby facilitating immobilization [128]. The process of artificial microalgal immobilization can replicate the ecological advantages associated with biofilms while simultaneously extracting nutrients from wastewater. Furthermore, the absence of immobilization presents a significant obstacle in extracting microalgal cells from treated wastewater, thereby restricting the effectiveness and applicability of microalgae-based wastewater treatment methods [129]. The physiological and physicochemical characteristics of microalgal cells can vary due to disparities in growth conditions between immobilized and suspended cultures. The utilization of resources and elimination of pollution differ between dispersed and aggregated microalgae cultures, as observed in the existing literature [124].

The aggregation of microalgal cells, either encapsulated in gel beads or attached to non-suspended carriers, results in a higher partial cell density than a system where the cells are freely suspended [130]. In the context of microalgae systems, it has been observed that immobilization techniques, coupled with an optimized structural arrangement and improved light utilization, can lead to a notable enhancement in cell growth potential and effective removal of pollutants [131]. Considering the green hydrogen derived from municipal wastewater via bioconversion by attaching microalgae onto various sizes of polyurethane foam cubes, Ardo et al. [132] stated that Polyurethane foam cubes of four different sizes were then added to the municipal wastewater medium to feed the connected microalgae that were performing the dark fermentation. The outcomes showed that the 1 cm cubes could consistently produce high hydrogen quantities of 20–21 mL, with COD and ammoniacal nitrogen removal efficiency obtained at 70% and 57%, respectively. Due to the high losses of connected microalgae caused by the abrasion process when the cubes were fluidizing in the culture media, cubes smaller than 1 cm failed to retain their hydrogen outputs due to the small surface area available to fill connected microalgae, larger cubes than 1 cm produced less hydrogen [132].

Additionally, there is a restriction on the amount of critical nutrients that can diffuse into the cubes from the culture medium to support the metabolic activities of connected microalgae inside the cubes. The first-order kinetics of the hydrogen production from 1 cm cubes was likewise well fit. In a study conducted by Rosales et al. [133], it was observed that the immobilization of *Scenedesmus* sp. in chitosan capsules resulted in greater efficiency in the removal of P, C, and N compared to a suspended system. According to a study conducted by Sánchez-Saavedra et al. [134], an immobilized system’s collective and collaborative effects can aid in the survival and adaptation of microalgal cells in the face of environmental challenges or toxicity. According to Nishi et al. [135], immobilization has effectively decreased the toxicity of inorganic metal oxide nano-adsorbents and carriers during the treatment of N and P wastewater. This immobilization technique also enhances the ability to resist disruption of cell development, prevents photo-inhibition, and reduces cellular toxicity. In addition, utilizing larger immobilized microalgal beads or immobilized carriers can enhance accessibility and reduce the energy intensity of conventional harvesting and dewatering techniques. Once more, the utilization of immobilized microalgal culture has the potential to mitigate the potential introduction of foreign microorganisms to the indigenous ecosystem [124]. This is due to the ability of the immobilizing beads to impede the release of the immobilized microorganisms into the wastewater [135]. Nevertheless, microalgal immobilization presents several drawbacks. According to Han et al. [122], the presence of polymers and carriers in immobilization systems can impede mass transfer and hinder resource absorption.

The effects of immobilized carriers and reagents on subsequent processing stages remain to be ascertained [136]. Confirming the stability and reproducibility of immobilized microalgae in actual wastewater treatment is imperative. Although microalgal biomass is known as “energy-rich waste,” attaining a workable energy balance in microalgal cultivation operations is extremely difficult since the microalgal biomass in dilute suspension cultivation is only about 0.02–0.05% dry weight [137]. Moreover, when comparing a suspended system to a system utilizing microalgal immobilization, it is important to consider the potential implications on operating costs and the demand for operational personnel [124]. A prolonged period of operation, in which the whole process relies on the ability to uptake and store nutrients of microalgae in wastewater, can have a notable impact, as it may lead to the release of microalgae, thereby causing subsequent environmental contamination and hazards. Hence, the utilization of immobilized microalgae presents a potential avenue for enhancing the efficacy of microalgae-based biological wastewater treatment, owing to their facile harvesting and enhanced environmental resilience.

The utilization of immobilized microalgae has the potential to be a viable approach for wastewater treatment [122]. Nevertheless, certain formidable obstacles hinder the development of a comprehensive, dependable, and economically viable microalgal immobilization system to treat actual wastewater. A pneumatic extrusion system consisting of multiple materials was successfully integrated into the end effector of a robotic arm [138]. This innovative approach enabled the fabrication of hydrogel membranes containing microalgae on a large scale. The findings of this study have significant implications for various industrial applications, particularly in the areas of microalgal bioremediation, bioenergy, and bioremediation, as it presents a commercially feasible method [138].

The term “Green Bioprinting” pertains to a method of immobilization explicitly developed for the 3D-bioprinting of microalgae, which exhibits remarkable viability and enhanced growth even in adverse temperature conditions [139]. The utilization of 3D printing to create silk protein hydrogels has been explored as a means to support the growth of microalgae [140]. Research has shown that these immobilized systems exhibit favorable characteristics (e.g., sustained cell viability, consistent photosynthetic activity, and exceptional cell performance) [137]. Consequently, these findings suggest that such systems hold promise in addressing the need for microalgae-based aquatic cleanup [141]. Additional support for the efficacy of inkjet printing in immobilizing microalgae and its ability to accurately control the size and quantity of encapsulated spores was presented by Lee et al. [142]. The researchers employed drop-on-demand inkjet printing to immobilize spores of *Ecklonia cav*a within alginate microparticles. Trampe et al. [143] used green microalgae as the bio-ink in conjunction with a chemical nano-sensor to track the cell metabolism and spatiotemporal dynamics of their chemical milieu in a 3D-printed structure. Immobilized microalgae's potential for large-scale production has been the subject of several studies, but its potential for use, particularly in wastewater treatment, has not yet been thoroughly assessed. When scaled up, immobilized microalgae could exhibit varying performances due to the complicated environment of real wastewater [144]. There are also a few effective on-site pretreatments offered. In a small reactor, microalgal beads immobilized in alginate were employed to remove nutrients from wastewater and make it easier to harvest the algae for biorefineries. A photo-rotating biological contactor inoculated with local microalgae was successfully scaled [145]. Various prevalent heavy metals and trace elements were effectively eliminated from a synthetic acid mine drainage containing multiple ions. Furthermore, swine wastewater treatment was effectively carried out by utilizing tubular chains consisting of immobilized *Dermocarpella* sp. in agar-alginate matrices [146]. Microalgal immobilization is gaining increasing attention as one of the most effective alternatives for upgrading conventional wastewater treatment systems.

## Phycoremediation of refractory pollutants

With industrial development, the amount of wastewater discharged is increasing daily, and removing refractory pollutants from wastewater has become one of the public’s main concerns [147]. Removing refractory organic pollutants (e.g., phenol compounds, pesticides, medical wastewater, synthetic dyes, and surfactants) is crucial because they are ubiquitous in the environment and seriously threaten ecosystems and human health [148]. The significance of enabling microalgae to acclimate to polluted environments prior to treatment is underscored by recent developments in algal bioremediation for refractory pollutants [149, 150]. In order to ensure that treatment is effective, it is essential to achieve optimal biomass levels prior to exposure. This entails the meticulous diluting of the contaminated solution to guarantee that it receives sufficient light penetration to facilitate mixotrophic benefits. Upon achieving an optimal development environment, microalgae implement numerous strategies to efficiently eliminate pollutants [87]. A prevalent process, surface adsorption is characterized by passive, spontaneous interactions and includes ion exchange and complexation with ionic pollutants. Bioaccumulation within algal cells is the consequence of progressive, energy-demanding bio-uptake, which follows rapid adsorption. Enzyme-mediated biodegradation and, in certain cases, photobiocatalysis occur within the cells [151].

In order to remediate a variety of pollutants, including emerging ones, microalgae utilize a variety of mechanisms, including adsorption, bioadsorption, bio-uptake, photodegradation, and biodegradation. The overall efficacy of bioremediation is influenced by the species and cell wall structures, which are the basis for these mechanisms [52]. Enzymes facilitate the conversion of selective pollutants into simpler, non-toxic forms during biodegradation, which involves their intracellular transport [5]. Bioadsorption, whether intracellular or extracellular, is facilitated by microalgal enzymes, which facilitate the degradation of organic and emerging pollutants into smaller, less toxic, and more stable molecules [152]. Algae have lately been the focus of extensive research regarding the mechanism of bioremediation, which has uncovered their immense potential as biological instruments for the remediation of polluted environments. The hazards to human and environmental health posed by the emissions of inorganic and organic chemicals from various industries have been addressed through the development of a variety of methods and technologies, including physical, chemical, and advanced oxidation procedures [153]. Although these techniques have the potential to generate new detrimental byproducts, they frequently have cost and efficacy constraints. Consequently, it is imperative to conduct research on practical and cost-effective technologies that offer long-term solutions, minimal chemical consumption, and safe end products.

### Industrial effluents

**Fig. 5:**
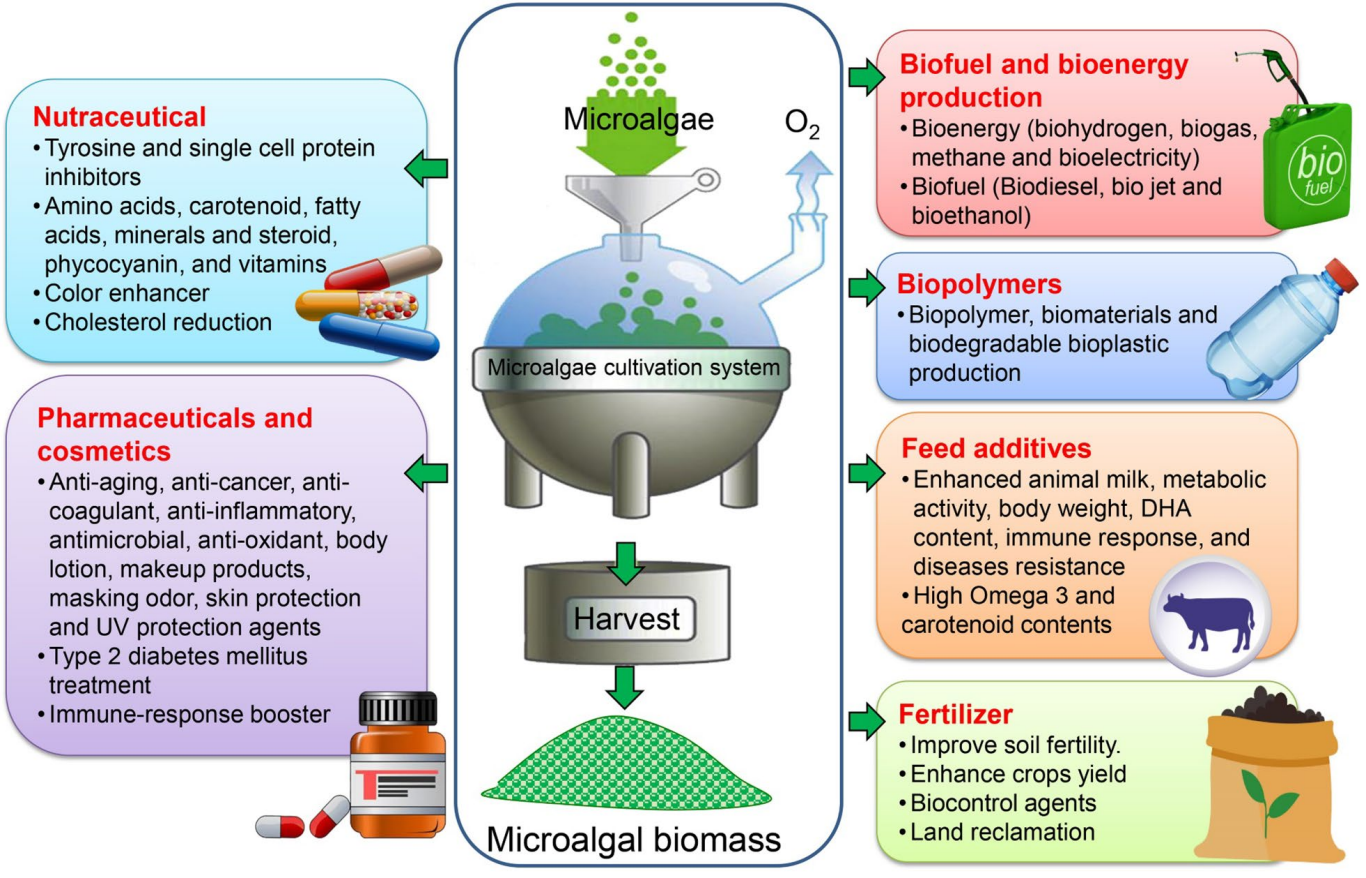
Microalgal cultivation and biomass harvesting for biorefinery applications

**Fig. 6:**
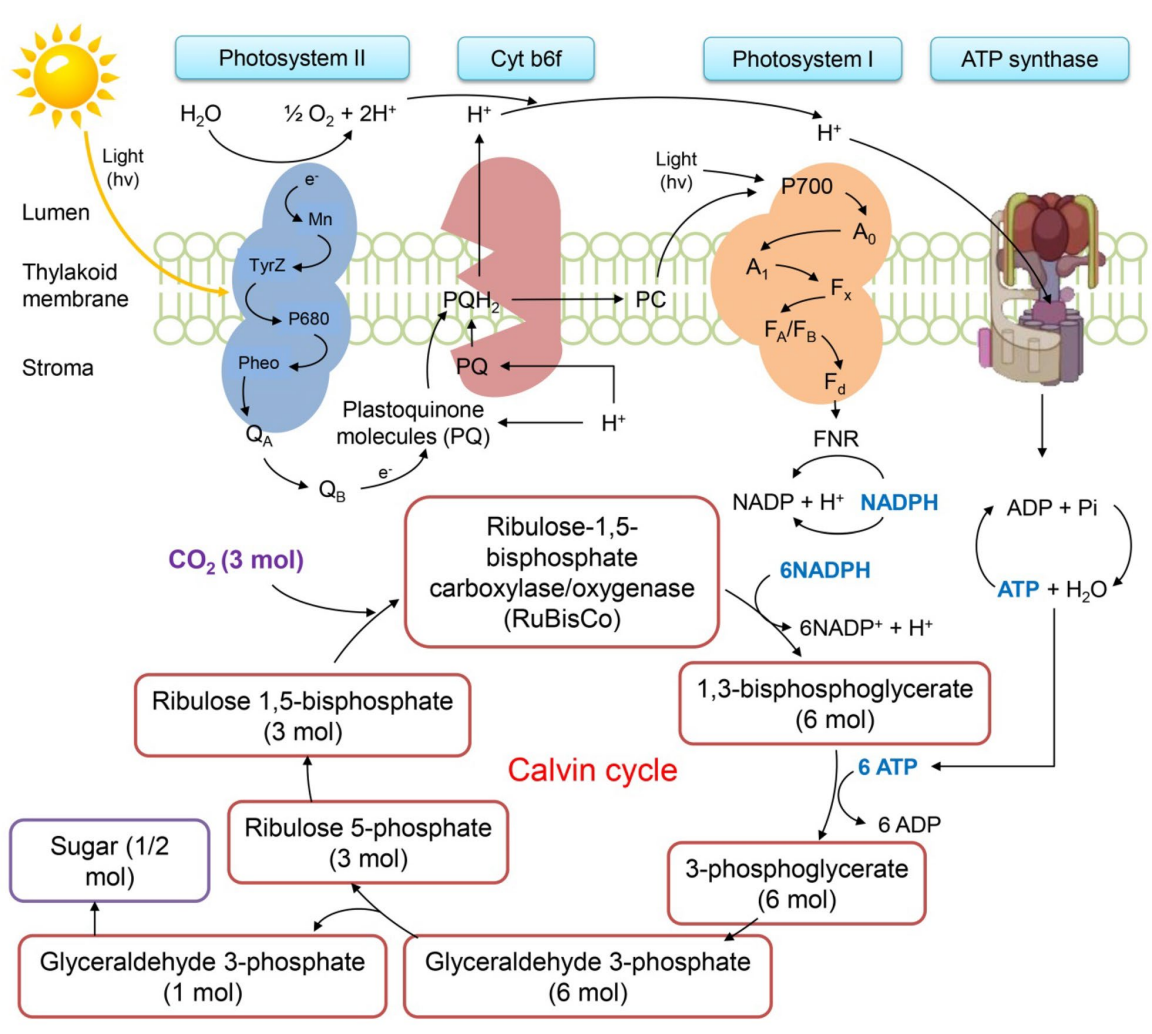
The major supercomplexes schematic diagram of microalgal metabolism for sugar biosynthesis

Using algae, contaminants can be removed from the environment or changed into harmless forms [154]. The biomass produced by algae is used to produce biogas, biofuel, and other high-value-added products, as shown in Fig. 5. Algal-based bioremediation is strongly favored due to its ability to raise biological oxygen demand (BOD) in contaminated water by fixing CO_2_ and releasing O_2_ through photosynthesis, as shown in Fig. 6. Efficient water treatment measures must be made to be safe and reduce our water bodies' toxicity [155]. Dahiya [156] stated that heavy metals like lead, cadmium, mercury, nickel, zinc, aluminum, arsenic, copper, and iron are severe environmental contaminants that lead to poisoning. Bioremediation is a pollution control method that uses biological systems to catalyze the breakdown or conversion of different chemicals into less dangerous forms. Economically less expensive and more environmentally friendly is thought to be the development of biologically based treatment systems [157].

For the pre-treatment of wastewater streams containing copper (II), *Padina* sp. biomass may be employed as an effective biosorbent. The maximum biosorption capacity was 0.08 m mol/g at a solution pH of around 5, and biosorption kinetics was found to be rapid, with 90% adsorption in 15 min [158]. The biosorption capacity of *Spirulina* was calculated to be 0.62 mg of lead per 105 alga cells during the initial stage (0–12 min), where its adsorption rate is so high that it physiologically adsorbs 74% of the metal. It was investigated how well-dried, dead *C. vulgaris* did at binding divalent Cu, Cd, and Pb ions from their aqueous solutions. In general, the percentage uptake of cadmium ions decreased as the dielectric constant values decreased. The percentage uptake of copper and lead ions decreased as the number of donor cells [159]. The levels of the BOD and chemical oxygen demand (COD) were decreased by *Scenedesmus obliquus* by up to 16.66% and 82.80%, respectively. BOD and COD levels may be reduced due to the treatment process's partial elimination of dissolved organic compounds and derivatives from effluents. *S. obliquus* treatment of refinery effluents is a method that successfully reduces pollutants [160]. *Oscillatoria* has been used to examine the efficacy of the Cyanobacteria treatment system for the bioremediation of textile effluents. 57.6% and 39.82% less COD and BOD, respectively [161]. Sousa et al. [162] concluded that using microalgae in biomonitoring, the phytoextraction and biodegradation of numerous organic contaminants is favored by restoring aquatic systems.

Other persistent organic pollutants are still difficult for the microalgae to degrade. This issue can be resolved through genetic engineering or nanotechnology applications, which also provide a method to promote the bioremediation of various organic contaminants, improve their absorption, and raise the microalga's resiliency to these pollutants [163]. Exploring and controlling several aquatic ecosystem characteristics (e.g., temperature, pH, nutrient availability, and other environmental parameters) are required to speed up the bioremediation process and shorten the decontamination time. To overcome the wastewater turbidity on photosynthesis of microalgae, the potential of artificial photosynthesis and microalgae to produce clean energy from renewable sources is part of a global push to combat fossil fuel use and slow climate change. Because microalgae naturally photosynthesize, they have proven that their biocompounds can generate clean energy while lowering their carbon footprint by absorbing carbon for growth. Microalgae that have been genetically altered have much better photosynthetic volume and cell growth [164].

Cultivation of *C. vulgaris* presents a maximum cellular concentration of Cmax and a maximum specific growth rate in wastewater between a 5% and 17.5% concentration [165]. The greatest COD and color removal happened in 17.5% of the cases of textile wastewater. The culture of *C. vulgaris* in Wastewater from textile waste revealed the potential for COD with this microalga and color elimination [165]. The level of COD and phenolic compounds in olive mill wastewater was effectively reduced by *C. vulgaris*, *S. platensis*, and *D. saline* [166]. Pesticides are used in agriculture to boost agricultural output while reducing crop loss. Pesticide toxicity in water increases as a result of agricultural discharge. When a pesticide is introduced into a body of water, it attacks species that are not intended targets, which upsets the aquatic life. Biological bioremediation techniques are typically selected due to their low cost of taking, high material removal efficiency, low sludgy amount, and generated biomass for economic advantage [167].

Microbial biomass serves as the primary feedstock for the manufacturing of numerous commodities. Consequently, using microalgae-based technology presents a diverse array of potential applications in both environmental and product development contexts [168]. Various challenges, including inadequate environmental adaptability, difficulties in recycling, and the potential for secondary contamination, accompany the use of non-immobilized biomass materials in practical applications. Biomass immobilization is an innovative technique for environmental remediation that effectively tackles these challenges. The utilization of immobilized biomass materials offers several advantages over non-immobilized biomass, including enhanced reusability and improved stability in pH, temperature, handling, and storage conditions. A considerable body of research has been dedicated to exploring immobilization technology, encompassing various techniques, carriers, and biomass types, with the primary objective of eliminating refractory organic contaminants [169].

The practical use of non-immobilized biomass resources has issues (e.g., inadequate environmental adaptation, recycling challenges, and secondary contamination). An innovative approach to environmental remediation that can successfully address these issues is biomass immobilization. Immobilized biomass materials are more reusable and stable regarding pH, temperature, handling, and storage than non-immobilized biomass materials. Numerous researchers have investigated immobilization technology’s potential for eliminating refractory organic contaminants, including its methodologies, carriers, and types of biomass [170]. On the other hand, Liu et al. [169] stated that immobilized biomass materials are more stable, more resistant to hostile environments, and have better recovery and reusability than free biomass. This technique is, therefore, a viable area for further study. The importance of biomass materials immobilized in environmental bioremediation was confirmed in this study, which covers the roles of immobilization techniques and biomass materials in immobilization technology and concentrates on the immobilization of biomass materials in organic wastewater treatment in recent years. A conclusion drawn from their results is that phycoremediation using several algae species, including *C. vulgaris* and *C. salina*, can recycle and reuse various mixes of water samples. The results of the current experiment demonstrated that both algae species had a very high capacity to lower the hazardous level of all physicochemical parameters. These studies demonstrate the effectiveness of *C. vulgaris* and *C. salina* as nutrient removers [171].

### Synthetic dyes

Global industrialization has exposed the biosphere to many potentially toxic, mutagenic, and xenobiotic compounds [172]. Several approaches must be used to identify effective, long-lasting solutions when removing harmful chemicals from the environment [173]. Unlike natural dyes, synthetic dyes are more cost-effective, long-lasting, and have a larger color spectrum. The untreated effluent from the textile sector is discharged into receiving water bodies in millions of liters daily, harming human health. A typical textile factory in India produces 60 × 10^4^ m of cloth daily, generating more than 1.5 million liters of wastewater [174]. Due to the increased discharge of toxins into the environment, rapid industrialization creates major environmental risks. Industries that release wastewater containing dye with or without pretreatment straight into water bodies are a major source of dye pollutants. This causes substantial water pollution in the environment. Therefore, it is crucial to protect the ecosystem against such toxins.

The traditional approach of treating wastewater contaminated with dyes is typically expensive and can potentially create secondary metabolites [175]. Due to the aforementioned issues, the biological technique is preferred to treat effluent or dye-contaminated wastewater. Several studies investigated the ability of microalgae to remove dye compounds from wastewater, as shown in Table 2 [176–189]. Getachew et al. [190] stated that phycoremediation is an algae-based eco-friendly dye abatement technology from contaminated areas. The authors also concluded that the advantages of phycoremediation technology (e.g., ease of availability, high efficiency, cost-effectiveness, large specific surface area, environmental friendliness, and chemical and physical stability), make it an essential tool, particularly in emerging and poor countries. Algae’s cell wall contains a variety of functional groups, including amino, carboxyl, hydroxyl, and phosphate groups, which are in charge of the dye removal process. The key factors for removing dyes include a variety of operational parameters (e.g., solution pH, contact time, initial dye concentration, adsorbent dosage, and temperature). Therefore, these criteria are considered when assessing how well algae perform in dye abatement. Although alga-based removal technologies have advanced, they still have significant limits that call for further investigation.

**Table 2:** Potential of microalgae to remove dyes from wastewater

Dye type	Dye conc. (mg/L)	Microalgal species	Incubation (days)	Removal rate (%)	References
Methylene Blue	10	*Chlorella vulgaris*	14	99.9	[176]
Methyl orange	10	*Bracteacoccus* sp.	NA	97	[177]
Crystal violet	10	*Arthrospira platensis* and *Spirulina* sp.	1	98.4	[178]
Methyl orange	10	*Arthrospira platensis* and *Spirulina* sp.	1	99	[178]
Reactive red 194	20 ppm	*Chlorococcum *sp. mixed with* Scenedesmus obliquus*	7	100	[179]
Reactive orange 122	40 ppm	*Chlorococcum *sp.	7	98	[179]
Malachite green	100	*Haematococcus* sp.	NA	67	[180]
Black dye	NA	*Gonium* sp. and microalgae mixture	15	40	[181]
Aniline blue	25	*Chlorella* sp.	11	58	[182]
Direct red 31	40	*Desmodesmus* sp.	14	90	[183]
Malachite green	5	*Oscillatoria* sp.	5	93	[184]
Methylene blue	5	*Oscillatoria* sp.	5	66	[184]
Safranin	5	*Oscillatoria* sp.	5	52	[184]
Methylene blue	100	*Chlorella vulgaris*	3	83.04	[185]
Yellow dye	10	*Chlorella vulgaris*	14	43.12	[186]
Direct blue 71	300	*Chlorella vulgaris*	12	78	[187]
Disperse red 1	200	*Chlorella vulgaris*	12	84	[187]
Reactive black 5	200	*Chlorella vulgaris*	12	80	[187]
Congo red	50	*Chlorella vulgaris*	9	100	[188]
Methyl red	20 ppm	*Scenedesmus obliquus*	10	48.60	[189]

Over the past three decades, the paper and textile industries have approved several physical, chemical, and biological decolorization processes [191]. In order to effectively remove colors from significant amounts of effluents, alternative biodegradations are needed (e.g., biological or combination systems) [192]. The ability to degrade colors has been seen in various microorganisms, including bacteria, fungi, yeasts, actinomycetes, and algae. Algae are tiny, photosynthesizing organisms that inhabit soil, water, and other open spaces. The researchers concluded that both algae have sufficient biodegradation capacity under ideal conditions to eliminate the colored pigments blue and red from their aqueous solution. *Spirogyra* sp. has been found to have a more promising ability for biodegradation than *Oscillatoria* sp. The results of this study are consistent with the idea that both kinds of algae can be used to eradicate blue-green algae to remove blue and red dye from waste effluents [193]*.*

Dye compounds in textile industry wastewater are resistant and challenging for biological processes to break down. The textile sector accounts for two-thirds of the markets for dyestuffs. About 10–15% of the color used during the dyeing process is discharged into the wastewater [194]. *Spirogyra* sp. and *Oscillatoria* sp. were investigated for their potential to biodegrade blue dye and red dye [191, 195]. Brahmbhatt and Jasrai [196] stated that decolorization testing, physicochemical examination, FTIR spectra, and UV spectrophotometry analysis were used to measure degradation. Products generated during degradation were also identified. The study also examined phytotoxicity and tested the toxicity of untreated and treated dye effluents. They concluded that both algae had adequate biodegradation potential for removing red and blue color from its aqueous solution [196].

There is a growing concern about algae’s ability to degrade textile colors because they are so common [7]. Many different kinds of algae have been successfully used in these techniques. Algae are more efficient as adsorbents for treating larger amounts of dye effluent than commercially available synthetic adsorbents because of their higher biomass content and capacity for biodegradation processes [197]. Algae degrade colors by three main methods, including (i) using the dyes for growth, (ii) converting the dyes into other intermediates such as CO_2_ and water, and (iii) adhering the chromophores to the algae [179]. In contrast to biodegradation, which is the process by which enzymes change one molecule into another, biosorption denotes the adsorption of pigments from water to solid phases (bio-adsorbents) [198]. It has been discovered that several *Chlorella* and *Oscillotoria* species can convert azo dyes into their aromatic amines, which can then be transformed into CO_2_ or simple organic compounds [199]. Microalgae may degrade industrial dye effluents and have been shown to have considerable azoreductase enzyme decolorizing activity, indicating its potential in a wide range of textiles and other products [200].

### Pharmaceuticals

Pharmaceuticals have been recognized as a significant class of environmental pollutants in recent years [201, 202]. Microalgae-based technologies have recently drawn increased interest, primarily because of their easy-growing requirements, ability to fix CO_2_, and minimal running expenses. Depending on the experimental settings, algae can switch from a heterotrophic to an autotrophic mode, allowing them to remove a variety of organic contaminants [203]. Additionally, they have a reputation for removing heavy metals and degrading xenobiotic chemicals. Microalgal systems are useful because they can recover resources from wastewater and simultaneously break down contaminants like antibiotics and pharmaceuticals while growing without the need for extra nutrients. The primary processes in pollutant removal by microalgae include bioadsorption, degradation, and accumulation [204]. Although microalgae-based systems seem promising and healthy alternatives for removing organic pollutants from wastewater systems, it is obvious that some issues must be resolved before the development of practical wastewater treatment methods can precede. The principal difficulties include the effectiveness of algal cultivation using actual wastewater, although the majority of studies have concentrated on one or a few small-scale pharmaceutical/organic pollutants [205]. Also, the composition of actual wastewater from industries and municipal sewage systems is more important and diversified [206].

Higher quantities of antibiotics may be harmful and fatal for the biotreatment process. Zhang et al. [207] reported that *C. pyrenoidosa* grows more readily in wastewater with lower concentrations of the contaminants carbamazepine, clofibric acid, ciprofloxacin, and diclofenac than when these pollutants are present in higher quantities. Therefore, the effectiveness of an algal-based treatment is difficult to anticipate because some contaminants and metabolites may prevent algae from growing and/or surviving. It is necessary to create more effective analytical techniques to find different pollutants in low quantities. Some medication compounds are hydrophobic and have very poor water solubility [208]. Some of the pharmaceutical pollutants with low solubility include celecoxib, ritonavir, doxorubicin, tamoxifen, herceptin, diclofenac, nitrendipine, and iso-liquiritigenin, which is used as a potent human monoamine oxidase inhibitor, modulates dopamine D_1_, D_3_, and vasopressin V_1A_ receptors [209]. These compounds separate from the water and are no longer available to the algae [208].

Advanced detoxification techniques like UV therapy, which can kill microbial pathogens without harming algae or other members of algal consortia, must be combined with biodegradation to reduce the likelihood of pathogen survival [210]. Due to their capacity to simultaneously handle several issues, algal-based treatment systems have enormous potential for wastewater treatment and environmental preservation. Challenges to their success must be properly addressed to build these systems into economically viable businesses [208].

## Nanotechnology for the bioremediation of refractory pollutants

**Fig. 7:**
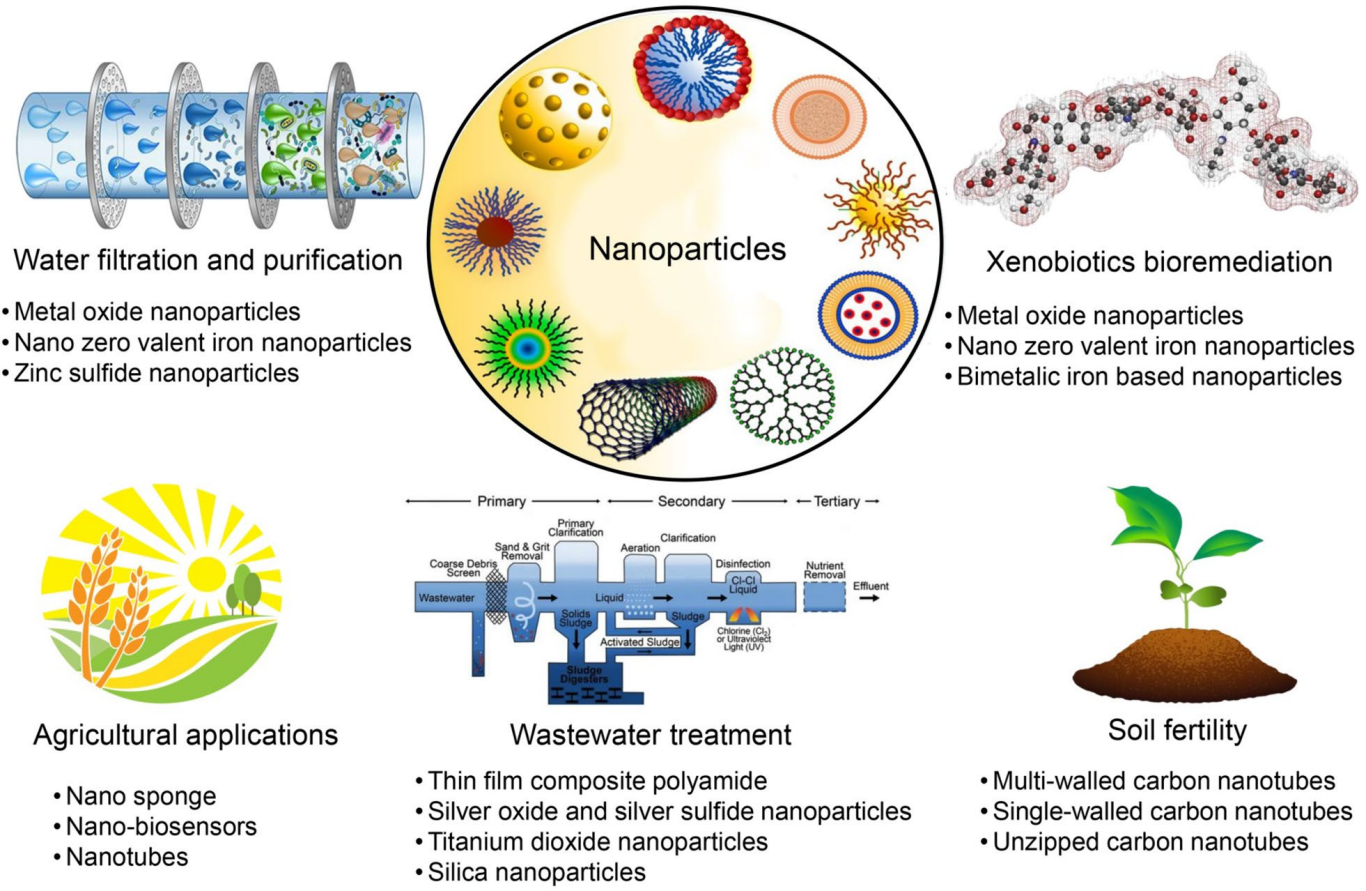
Various applications of nanoparticles

Nanotechnology has the potential to substantially contribute to the advancement of cleaner, more environmentally friendly technologies that offer significant health and environmental benefits [211]. The potential of nanotechnology techniques to enhance the performance of conventional environmental clean-up methods and to provide solutions for pollution management and mitigation is currently being investigated [212]. Nanotechnology has the potential to have a positive impact on the environment by lowering the amount of energy that is consumed during the production process, making it easier to recycle products after they have been used, and encouraging the creation and utilization of materials that are friendly to the environment (Fig. 7). However, it is vital to take into account the negative implications that nanotechnology has on human health and the environment [213]. Nanotechnology now has the potential to address significant difficulties that are associated with sustainability [214]. The emphasis on addressing modern refractory pollutants has only recently emerged, despite the fact that microalgae's ability to remediate organic and inorganic contaminants has been recognized for over thirty years [152]. By removing persistent pollutants, the cost-efficiency of wastewater treatment is enhanced and environmental conservation is facilitated by the integration of refractory pollutant bioremediation and nutrient removal by microalgae (Fig. 7). By extracting pollutants from air, soil, and water, microalgae are crucial in the mitigation of environmental pollution [215].

Over the course of the last several years, there has been a substantial rise in the manufacture of nanoscale items that are designed for the purpose of environmental remediation [216]. Nanomaterials have been utilized in the remediation of contaminated soil and groundwater at a number of hazardous waste sites, including those that have been damaged by chlorinated solvents and oil accidents (Fig. 7). The physicochemical, surface, and optical-electronic features of tailored nanoparticles have the potential to solve problems that were previously difficult to tackle using procedures that were considered to be conventional [217]. In addition to the ability to produce high-performance materials and chemicals with little energy consumption, it is also capable of developing novel ways for the development of new techniques, the substitution of existing instruments, and the manufacturing of new techniques [218]. When nanoparticles and microalgae interact, the elimination of contaminants is not the only thing that may be accomplished by this interaction [219]. They not only throw light on the production stage, but they also play an important part in the bioremediation process as a whole. In order to improve bioremediation efforts and to increase the efficiency and effectiveness of microalgae production, nanoparticles offer a diverse approach to sustainable environmental management [220].

In the field of bioremediation, microalgae that have been augmented with nanoparticles that are specifically targeted provides a viable option [221]. According to the treatment situation and the characteristics of the nanomaterial, nanomaterials can perform a range of activities, including those of nanocatalysts, nanoadsorbents, nanophotocatalysts, and nanomembranes [222, 223]. This allows them to demonstrate their effectiveness in remediation. Nanoparticles have certain qualities that make them favorable for use in biological applications. These properties include biocompatibility, chemical stability, and supermagnetic capabilities [224]. An additional noteworthy characteristic of nanoparticles is their capacity to interact with microalgae cells, which are typically 5–10 μm in size, due to the nanoscale diameter of the apertures in the cell walls [225]. Nanoparticles initially adhere to the microalgal surface and subsequently accumulate within the cells through active transport, which is influenced by the negatively charged algal cell wall [226]. It is believed that algae accumulate heavy metals, which could be utilized in the biogenic production of metallic nanoparticles [227]. *C. vulgaris* was able to produce nanoparticles that gathered toward the surface. These nanoparticles were tetrahedral, decahedral, and icosahedral in shape. The proteins that are present in the algal extract serve a variety of purposes, including those of a stabilizing agent, a reducing agent, and a shape-control modifier [228]. The marine alga *Sargassum wightii* is responsible for the production of extracellular nanoparticles of silver and gold [229]. Due to the fact that they are formed from living *Euglena gracilis* cells that have been grown under either mixotrophic or autotrophic conditions, the gold nanoparticles that are produced are of a real yield [230]. Compared to traditional methods, numerous NPs or NMs have demonstrated significant efficacy in removing diverse environmental toxins, as evidenced by the findings presented in Table 3 [231–243].

**Table 3:** Applications of algal silver nanoparticles

Silver-synthesized algae	Applications	References
*Asterarcys* sp.	Potent antibacterial and antifungal activities against the pathogens. Also, photocatalytically degrade hazardous dye methylene blue	[231]
*Arthrospira platensis*	Strong antibacterial effect against bacteria isolated from human urinary tract infections	[232]
*Oedogonium* sp., *Ulothrix* sp., *Cladophora* sp., and *Spirogyra* sp.	Enhance metabolite accumulation and biodiesel production	[233]
*Spirulina *sp.	Potent antimicrobial against multi-drug resistant pathogens	[234]
*Dunaliella salina*	Antibacterial activity	[235]
*Sargassum subrepandum*	Anticancer, antimicrobial, and molluscicidal activities	[236]
*Spirulina platensis*	Anticancer, antibacterial, and free radical scavenging capabilities	[237]
*Chlorella vulgaris*	Photocatalytic dye degradation activity	[238]
*Caulerpa serrulata*	Antimicrobial activity	[239]
*Chlorella vulgarts*	Degrade polycyclic aromatic hydrocarbons	[240]
*Microchaete* sp. NCCU-342	Degrade azo dye methyl red	[241]
*Scenedesmus quadricauda*	Potent antibacterial and antifungal activities against the pathogens	[240]
*Selenastrum capricornutum*	Degrade polycyclic aromatic hydrocarbons	[240]
*Scenedesmus platydiscus*	Degrade polycyclic aromatic hydrocarbons	[240]
*Spirulina platensis* and *Oscillatoria* sp.	Antiviral	[242]
*Sargassum latifolium*	Antibacterial	[243]

It has been argued that nanotechnology exhibits enhanced efficacy and cost-effectiveness, positioning it as a promising remediation technology for the future [244]. Using biodegradable materials presents a compelling prospect for this field of study. In addition to providing customers with a secure and ecologically conscious alternative, biodegradable products contribute to the mitigation of waste generation. Implementing target-specific detoxification techniques for pollutants has also bolstered the enhancement of cleanup methodologies. Integrating surface-modified NMs with novel techniques has been employed to effectively tackle the difficulties associated with eliminating pollutants [245].

The removal efficiency is enhanced by the increased surface area for pollutant adsorption that nanoparticles provide [246]. They also aid in the degradation of pollutants by generating reactive oxygen species, which are more effective at breaking down contaminants. In addition, nanoparticles can improve the metabolic activity of microalgae, resulting in a greater absorption and assimilation of pollutants [247]. Nevertheless, additional research is required to gain a comprehensive understanding of these interactions. The utilization of nanostructures and nanocomposites in water treatment has witnessed a significant rise due to their cost-effectiveness, lack of toxicity, and environmentally friendly nature [248]. Microalgae exhibit significant potential as a viable option for synthesizing NPs due to their ability to effectively reduce metal ions. The synthesis of microalgal-NPs has considered various factors (e.g., environmental conditions, kinetics, and the mechanism for NPs formation) [249]. These factors include the pH of the reaction mixture, selection of appropriate strains, ionic strength, light intensity, and the biological transition of metal cations [240]. Table 4 enumerates the benefits and drawbacks associated with microalgal-NPs. Numerous methodologies have been developed to fabricate metallic microalgal nanoparticles featuring nanocrystals of superior quality [230]. The production of silver nanoparticles (Ag-NPs) involved the utilization of cell-free culture supernatants derived from cyanobacteria and Chlorophyta species [250]. The biomolecules obtained from the disrupted microalgal cells can be effectively utilized. According to El-Sheekh and El-Kassas [250], the authors asserted that the synthesis of Ag-NPs can be achieved using *Spirulina platensis* and *Lyngbya majuscula* as starting materials. In a study conducted by Mahdieh et al. [251], it was found that *C. vulgaris* has the potential to serve as a viable source for synthesizing gold nanoparticles (Au-NPs). Furthermore, Agarwal et al. [240] asserted that microalgae can be utilized to synthesize palladium nanoparticles (Pd-NPs).

**Table 4:** Advantages and disadvantages of microalgal-NPs

Advantages	Disadvantages
Eco-friendly mitigation approach	Initial outlay or implementation costs are quite high
As technology users become more environmentally aware, microalgal-NPs gain popularity. In some cases, this will help investors in the long run	People are still learning the technology; therefore, it will take some time to embrace NPs applications
Long-term applications may decrease operational costs	Many products are still in the research and development stages since technology is still developing. Therefore, people are unaware of performance consequences
Uses renewable natural resources	Lack of qualified human resources to install or execute products or systems based on green technology
Decrease global warming impact due to microalgal-NPs applications due to reduction of CO_2_ emissions	NPs applications policies have not been finalized for microalgal-NPs

Nanofibers find utility in diverse applications, encompassing air filtration, template self-assembly in layered materials, and microsensors [252]. Nanofibers possess unique physicochemical and optical properties. To address the pollution issue in wastewater, combining nanofibers with biomacromolecules is possible [253]. The study conducted by Wang et al. [254] investigates the utilization of algae in combination with TiO_2_/Ag NMs to remove Cr (IV) under UV irradiation. The author found that the bio-nano hybrid materials maintained a high efficiency easily. These results gave enlightenment to employ these bio-nano hybrid materials to remove organic/inorganic contaminants from wastewater under irradiation. The study conducted by San Keskin et al. [255] investigated the removal of reactive dyes from water by utilizing a hybrid system consisting of polysulfone nanofibers and *Chlamydomonas reinhardtii*.

Because of the limitations and drawbacks of traditional immobilization techniques, alternative immobilization methods have been developed [256]. Immobilization can also be accomplished by combining two techniques: entrapment followed by covalent fixing or entrapment followed by cross-linking [257]. For pesticide detection, microalgal cells are immobilized by double encapsulation by encapsulating in alginate beads surrounded by silica hydrogel. When developing a sensitive biosensor, biological and physio-chemical characteristics must be considered. Double encapsulation is safe for the encapsulated cells and does not affect their growth [258]. Agarwal et al. [240] immobilized *C. rienhardtii* on polysulfone nanofiber to mitigate reactive metal dyes from wastewater; electrospun chitosan nanofiber mats was used to immobilize microalgal cells for nitrate removal from wastewater. To detect nano-encapsulated atrazine, *C. rienhardtii* was immobilized on an agar hydrogel/paper substrate [259]. Microalgae can be immobilized to detect atrazine in water using electrodes printed with polyelectrolyte-surfactant-carbon nanotubes [260]. In order to extract nitrogen and phosphorus from the secondary effluent, it was also possible to encapsulate *Chlorella vulgaris* on a membrane bioreactor [261]. Additionally, to extract the lipids from *Nannochloropsis* sp., cellulase, and lysozyme were co-immobilized on the surface of amino-functionalized magnetic NPs [262]. Particularly the immobilization of magnetic NPs; this field of study is still highly active and seems promising [256].

The delicate and crucial biosensor manufacturing process can impact the device's essential operational characteristics, including sensitivity and stability [263]. Therefore, selecting a support material is necessary in creating a biosensor. The support material must be stable, non-toxic, inexpensive, transparent, insoluble, and non-biodegradable. The biological system of cells is poisonous to NMs, which limits their application. NMs were discovered to be excellent supports for microalgae immobilization because of their high surface area, non-porous composition, high surface reactivity, super-paramagnetic properties, and high adsorption capacity to many ions [264]. Nevertheless, nanotechnology has been used in a wide variety of applications. Graphene, magnetic NPs, carbon nanotubes, nano-silica, noble-based NMs, metal, nanospheres, mesoporous silica NPs, and paper-based and fluorescent nanocrystals are a few examples of NMs that have been employed [265]. Noble and metal-based NPs, including gold, silver, platinum, iron, copper, palladium, cobalt, and metal oxides like ZnO and TiO_2_, are all used in NPs-based biosensors. Excellent optical, electrical, magnetic, chemical, catalytic, and mechanical properties may be found in these elements. Carbon nanotubes are hollow cylindrical tubes with fullerene hemispheres encasing one, two, or more concentric graphite layers. In addition to their distinctive structure, carbon nanotubes have innovative electron transport capabilities, a high surface-to-volume ratio, excellent electrical and mechanical properties, chemical stability, high thermal conductivity, and minimal surface fouling [263].

Ferrous ferric oxide (Fe_3_O_4_)-NPs are one of the materials used in biosensors. They are utilized in Surface Plasmon resonance-based biosensors, where Fe_3_O_4_-NPs are produced using the co-precipitation method. Once the Fe_3_O_4_-NPs are produced, they are functionalized with polyethylene glycol to adhere to microalgae [266]. By employing various support materials and biosensor types, *C. rienhardtii* could detect contaminants like atrazine. It was supported by electro-optical, amperometric, and optical biosensors, as well as paper-based screen-printed electrodes nanomodified with carbon black, carbon black-modified screen-printed electrodes, and hydrophilic quantum dots NPs, respectively [267]. Additionally, Kashem et al. [268] found that *Pseudokirchneriella subcapitata* was supported by a biosensor chip in a live algal biosensor that included oxygen arrays to detect several pesticides (e.g., simazine, atrazine, diruon, and simetryn). Additionally, *Monoraphidium contortum* has been immobilized carbon nanotubes in portable biosensors for atrazine detection [260]. Hence, additional research is necessary to explore the capacity of microalgal-NPs to address environmental contaminants and safeguard human health against various pollutants.

There is a possibility that bioremediation techniques based on microalgae could remove contaminants from effluent in a manner that is environmentally friendly; however, the implementation of these techniques on a broad scale is hampered by significant barriers [152]. Microorganisms, pharmaceuticals, organic contaminants, metals, and personal care products are all examples of the various composition of microplastics that can be found in wastewater [269]. This offers a substantial issue. Furthermore, the accidental retention of algal cells inside the system as a result of common processes such as filtration, sedimentation, centrifugation, and flotation might be a source of environmental concerns for aquatic ecosystems [270]. It is absolutely necessary to overcome these challenges in order to ensure that the bioremediation process that is based on microalgae will be successful.

A testament to the extraordinary progress that has been made in the subject is the current state of nano-enabled microalgae-assisted bioremediation, which is a state that is now in existence [271]. This ability of nanomaterials to improve the efficiency of bioremediation and to widen the spectrum of environmental uses of microalgae is a demonstration of the versatility of nanomaterials. A new era of environmentally responsible bioremediation procedures has been ushered in thanks to the incorporation of nanomaterials, which has resulted in a large rise in the pace at which pollutants are removed [271, 272]. Despite these challenges, there is a wealth of untapped potential in the field of microalgal wastewater treatment technology about the future. Advancing microalgae-based bioremediation sustainably requires fostering collaborations across academics, industry, and government entities, alongside technological innovations [273]. On a broader scale, these collaborations can enhance information transfer, expedite innovation, and streamline the adoption of sustainable solutions. The effective execution of microalgae-based bioremediation projects relies on the involvement of local communities and stakeholders to obtain support, address concerns, and acquire essential resources.

## Conclusion

Green technology has become increasingly prevalent in recent years due to its significant environmental benefits. Furthermore, wastewater contaminated with inorganic nitrogen, phosphorus, persistent organics, and refractory pollutants is discharged into the environment, leading to enduring problems. Costs associated with physical–chemical wastewater treatment typically rise, especially in developing countries. Conventional approaches to mitigating environmental pollution tend to consume more energy, while using certain approaches can lead to the generation of secondary forms of pollution; thus, these techniques may not be sustainable. Hence, there is a need for an alternative green, sustainable approach to efficiently treat wastewater. Recently, researchers have drawn attention to the potential of microalgae due to its cost-effectiveness, environmentally friendly nature, and ease of implementation. Moreover, microalgae have demonstrated their ability to undergo biotransformation and degradation of various refractory pollutants. Therefore, microalgal treatment for wastewater has been established as a feasible, sustainable biotechnological method for remediation. One more benefit of using microalgae to clean wastewater is that their biomass can be used to make a wide range of sustainable biofuels and bioenergy in circular bioeconomy applications. This makes them a good choice for tertiary biotreatment. On the other hand, this review highlights the importance of immobilizing microalgae as a viable solution to the harvest problem, while also retaining the high-value microalgal biomass for further processing. Moreover, nanotechnology holds significant potential as a viable solution to address various ecological challenges. The properties of bulk materials undergo significant changes when manipulated at the nanoscale, resulting in the ability to exhibit targeted effects against particular pollutants. Desirable properties enhance nanomaterials, including tunable surface functions, structural stability, high adsorption capabilities, improved selectivity and specificity, improved biodegradability, and increased reusability. The advantages of microalgae and their applications can inspire researchers to explore the biodegradation mechanism of different microalgal strains and novel techniques for microalgae immobilization, as well as the application of microalgae-based nanotechnology for efficient and sustainable bioremediation processes.

## Data Availability

No datasets were generated or analysed during the current study.
